# Drooling in Parkinson’s Disease: Prevalence and Progression from the Non-motor International Longitudinal Study

**DOI:** 10.1007/s00455-020-10102-5

**Published:** 2020-03-04

**Authors:** Daniel J. van Wamelen, Valentina Leta, Julia Johnson, Claudia Lazcano Ocampo, Aleksandra M. Podlewska, Katarina Rukavina, Alexandra Rizos, Pablo Martinez-Martin, K. Ray Chaudhuri

**Affiliations:** 1 Institute of Psychiatry, Psychology & Neuroscience, Department of Basic and Clinical Neurosciences, King’s College London, De Crespigny Park, London, SE5 8AF UK; 2 Parkinson Foundation Centre of Excellence At King’s College Hospital, Denmark Hill, London, SE5 9RS UK; 3 Donders Institute for Brain, Cognition and Behaviour, Department of Neurology, Radboud University Medical Centre, Nijmegen, the Netherlands; 4 Department of Neurology, Hospital Sotero del Rio, Santiago, Chile; 5 Center for Networked Biomedical Research in Neurodegenerative Diseases (CIBERNED), Carlos III Institute of Health, 28031 Madrid, Spain

**Keywords:** Parkinson’s disease, Drooling, Dysphagia, Non-motor symptoms scale, Deglutition, Deglutition disorders

## Abstract

Sialorrhoea in Parkinson’s disease (PD) is an often neglected yet key non-motor symptom with impact on patient quality of life. However, previous studies have shown a broad range of prevalence figures. To assess prevalence of drooling in PD and its relationship to quality of life, we performed a retrospective analysis of 728 consecutive PD patients who had a baseline and follow-up assessment as part of the Non-motor International Longitudinal Study (NILS), and for whom drooling presence and severity were available, assessed through the Non-Motor Symptoms Scale (NMSS). In addition, we analysed the prevalence of associated dysphagia through self-reported outcomes. Quality of life was assessed through the PDQ-8 scale. Baseline (disease duration 5.6 years) prevalence of drooling was 37.2% (score ≥ 1 NMSS question 19), and after 3.27 ± 1.74 years follow-up, this was 40.1% (*p* = 0.17). The prevalence of drooling increased with age (*p* < 0.001). The severity of drooling, however, did not change (*p* = 0.12). While in 456 patients without drooling at baseline, only 16% (*n* = 73) had dysphagia (question 20 of the NMSS), in those with drooling this was 34.3% (*p* < 0.001). At follow-up, the number of patients with dysphagia had increased, 20.4% with no drooling had dysphagia, and 43.6% with drooling had dysphagia. Both at baseline and follow-up, drooling severity was significantly positively associated with quality of life (PDQ-8; *r* = 0.199; *p* < 0.001). In moderately advanced PD patients, subjective drooling occurs in over one-third of patients and was significantly associated with decreased quality of life. Dysphagia occurred significantly more often in patients with drooling.

## Introduction

Drooling, or sialorrhoea, is a frequent non-motor symptom in Parkinson’s disease (PD). A wide prevalence range has been reported in the literature, ranging from 10 to 84% [1–5], and drooling has a negative impact on both patients and caregivers and is rated by patients as one of the top 10 most bothersome symptoms [6, 7]. The broad range is likely due to the lack of a standard definition and diagnostic criteria, and differences in the studied PD populations [5]. Drooling in PD appears to be primarily related to reduced swallowing efficiency, and not to an increase in saliva production [8]. Further contributing factors are reduced lip seal as part of hypomimia, sensory and postural changes and poor awareness of drooling [9]. Moreover, sialorrhoea is more prevalent in patients with cognitive impairment [10, 11].

Although many contributing factors to sialorhoea have been identified in PD, the exact cause of sialorrhea has not been fully clarified. The strongest evidence is that for a relationship between dysphagia and drooling [1, 12]. Other studies have shown that, based on measures of salivation [13–15], and scintigraphic analysis [16], impaired swallowing is the most likely cause of drooling in PD. The importance of drooling in PD has been demonstrated not only by the associated risk of dry mouth, impact on bolus formation, loss of antibacterial effects of saliva, perioral dermatological changes, dehydration, and increased speech difficulties [8, 17, 18], together with its social impact and associated social isolation. As the current literature in drooling in PD is often conflicting, we aimed to expand the current knowledge by assessing sialorrhoea and associated symptoms in a large cohort of longitudinally followed PD patients. The aim of the current study was to examine the prevalence of drooling, and its progression, across different age groups in patients with PD, and assess the impact on quality of life. Our hypothesis was that the prevalence of sialorrhoea in PD would increase with age and would negatively impact on quality of life.

## Methods

This study was a retrospective project using data from the prospective, longitudinal, Non-motor International Longitudinal Study (NILS). The NILS Study enables the charting of the natural history of non-motor symptoms together with treatment response and clinico-pathological correlations in PD patients. The study is adopted as a national study by the National Institute of Health Research in the UK (UKCRN No: 10,084) and involves 30 centres around the world, currently containing data for over 1600 PD patients who had a baseline assessment, and for some of whom a follow-up of up to seven years is available. The study has been authorised by local ethics committees (NRES SouthEast London REC3, 10,084, 10/H0808/141). All patients gave written consent prior to study procedures in accordance with the Declaration of Helsinki. The main inclusion criterion was a diagnosis of idiopathic PD according to the UK Brain Bank criteria. Exclusion criteria were (1) diagnosis of Parkinsonism different to idiopathic PD; (2) dementia (MMSE scores of 26 or less [19]); (3) inability to give informed consent to participate in the study.

For the current analysis, we used data from patients clinically diagnosed with idiopathic PD and whose data were entered between November 2011 and July 2019 and who were included in one of the UK centres for NILS. Data for analysis were extracted on July 1st 2019. Only patients who had at least one follow-up assessment as part of the NILS study were included in the analysis, and data for the last available follow-up assessment were used for analysis. Data extracted from the NILS database concerned sex, age, disease onset and duration (in years), and Levodopa equivalent daily dose (LEDD). Patient-reported outcomes included Hospital Anxiety and Depression Scale (HADS) [20], a quality of life measure (PDQ-8) [21], Parkinson’s Disease Sleep Scale-version 1 (PDSS) [22], Mini Mental State Examination (MMSE) [23], and Epworth Sleepiness Scale (ESS) [24] scores. Clinician-based evaluations included Hoehn and Yahr (HY) staging [25], Scales for Outcomes in PD [26], and Non-Motor Symptoms Scale (NMSS) and Questionnaire (NMSQ) scores [27, 28]. The NMSS categorises the frequency and severity of the non-motor symptoms of PD by nine domains: cardiovascular, sleep/fatigue, mood/apathy, perceptual problems/hallucinations, attention/memory, gastrointestinal tract, urinary function, sexual function, and miscellaneous [28].

The presence and frequency of drooling in the studied cohort was based on the scores for question 19 of the NMSS, asking about the presence and frequency of drooling. Using NMSS question 19 scores we stratified participants based on severity of drooling: (1) absent to mild (scores 0–3), (2) moderate (scores 4–7), and severe (scores 8–12). In addition, drooling was scored as either absent (score 0 on question 19 of the NMSS) or present (scores 1–12). Subsequently we assessed prevalence, and progression of drooling and drooling severity in our cohort. Secondary outcomes included a linear regression analysis to assess baseline predictors for drooling severity at follow-up. To this end, the raw scores for NMSS question 19 scores were used as a dependent continuous variable, and baseline demographics, and specific non-motor (HADS, PDSS, MMSE, ESS) and motor characteristics (SCOPA) as independent continuous variables. As the data were not normally distributed (Shapiro–Francia test), group differences at baseline and follow-up were tested with Mann–Whitney *U* test or Kruskal–Wallis test, where appropriate. To test for gender differences, Pearson Chi-square analysis was used. To test for binominal differences in baseline and follow-up drooling and dysphagia prevalence we used the McNemar test, and to test baseline to follow-up difference for continuous variables we used the Wilcoxon signed-rank test. For correlations Spearman’s ρ was used. All data were analysed using SPSS Version 25 (IBM SPSS Statistics for Windows, Version 25.0. Armonk, NY: IBM Corp.). Data are represented as mean  ± standard deviation, unless otherwise specified; a *p* value of < 0.05 was considered significant.

## Results

Of the 1120 participants who had a baseline assessment as part of the NILS study, 796 had at least one follow-up assessment. Of these participants, 728 had NMSS scores for question 19 (drooling) available for both baseline and follow-up analysis. Mean duration of follow-up for this cohort of 728 PD patients was 3.27 ± 1.74 years (range 0.5–7.2 years). Other baseline demographic data are presented in Table 1.

**Table 1:** Demographic data for the 728 participants for whom drooling scores were available at the baseline and last follow-up assessments

	Baseline	Follow-up	*p* value
Gender (M/F)	462/265 (63.5%/36.4%)	462/265 (63.5%/36.4%)	N/A
Age (yrs)	65.72 ± 10.87	68.98 ± 10.77	< 0.001
Disease duration (yrs)	5.63 ± 5.08	8.90 ± 5.37	< 0.001
Hoehn and Yahr stage	2.19 ± 0.89	2.58 ± 0.90	< 0.001
LEDD (mg)	525.19 ± 462.01	712.21 ± 468.78	< 0.001
SCOPA scores			
Motor score	9.85 ± 5.11	11.19 ± 5.49	< 0.001
Activities of daily living	5.29 ± 3.40	7.02 ± 4.00	< 0.001
Motor complications	1.68 ± 2.53	2.39 ± 2.41	< 0.001
NMSS total scores	45.48 ± 36.47	48.93 ± 39.38	0.012

Duration of follow-up was 3.27 ± 1.74 years (range 0.5–7.2 years)

*F* female, *LEDD* levodopa equivalent daily dose, *M* male, *NMSS* non-motor symptoms scale, *yrs* years

**Fig. 1:**
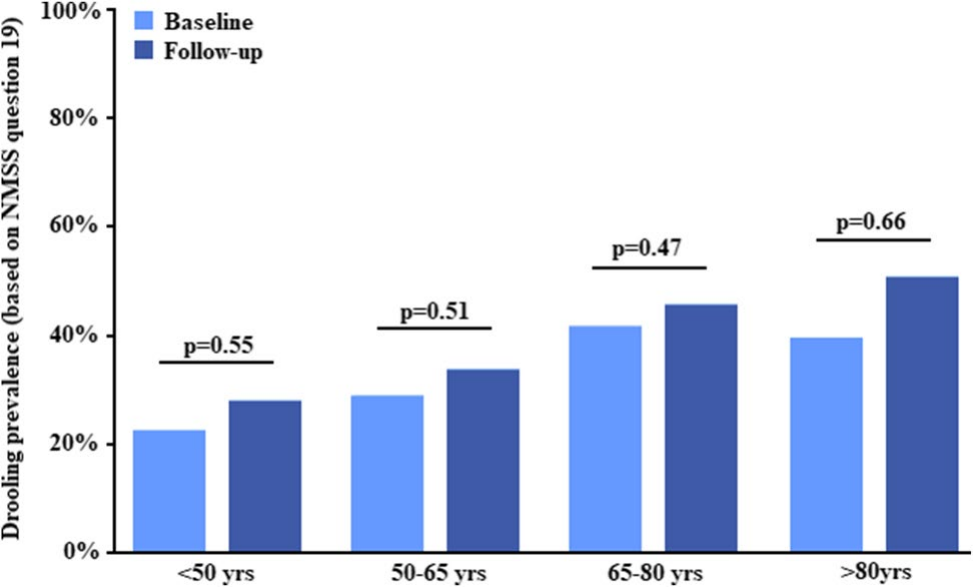
Prevalence of drooling, as defined by question 19 of the Non-Motor Symptoms Scale, at baseline and follow-up across four different age groups. *NMSS* non-motor symptom scale, *yrs* years. Duration of follow-up was 3.27 ± 1.74 years (range 0.5–7.2 years)

**Fig. 2:**
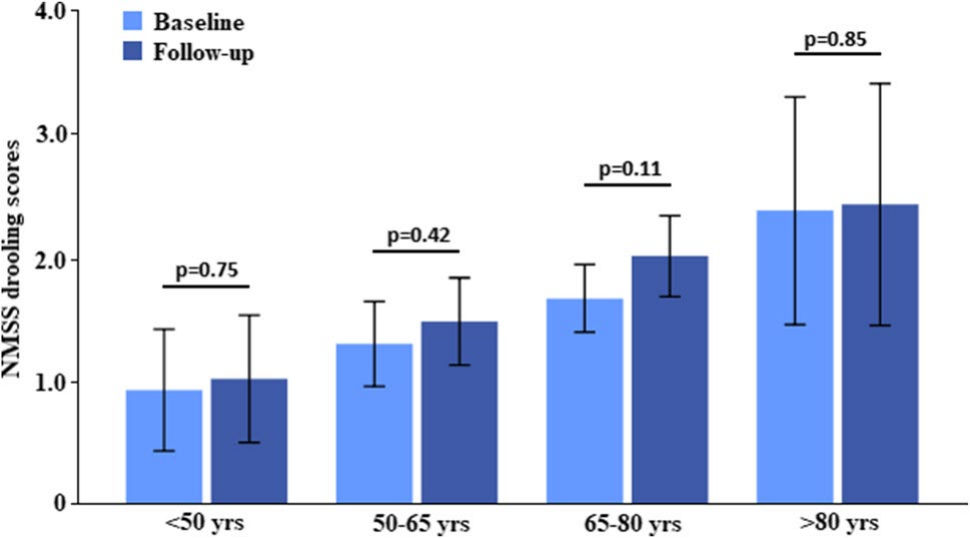
Drooling severity, as defined by question 19 of the Non-Motor Symptoms Scale, at baseline and follow-up across four different age groups. *NMSS* non-motor symptom scale, *yrs* years. Bars represent 95% confidence intervals

At baseline prevalence of drooling was 37.2% (score of  ≥ 1 on NMSS question 19), and after the 3.27 ± 1.74 years follow-up this the prevalence had remained stable at 40.1% (McNemar test; *p*  = 0.17; Table 2). Similar results were observed across the respective age groups, with the lowest prevalence (24.2%) in the youngest (< 50 years of age) patients, and the highest (45.8%) in the older patients (> 80 years of age) (Fig. 1; Table 2). The prevalence of drooling increased with age, in all four age groups at baseline and follow-up (Wilcoxon test; *p*< 0.001 and *p* = 0.002, respectively; Fig. 1; Table 2), and drooling severity (as measured by NMSS question 19) showed a significant, yet almost negligible, association with age, both at baseline (Spearman’s test; ρ = 0.134; *p* < 0.001) and at follow-up (Spearman’s test; ρ  = 0.159; *p* < 0.001). The severity of drooling did not change over the follow-up period, with mean scores of 1.53 ± 2.71 at baseline, and 1.77 ± 3.02 at follow-up (Wilcoxon test; *p* = 0.12). Subgroup analysis revealed that the latter was similar across all four age groups (Wilcoxon test; *p* ≥ 0.11; Fig. 2).

**Table 2:** Prevalence of drooling (based on Non-Motor Symptoms Scale question 19) at baseline at follow-up

Drooling	Baseline	Follow-up	*p* value
Entire cohort (*n* = 728)	271 (37.2%)	292 (40.1%)	0.17^a^
Age < 50 years (*n* = 66)	16 (24.2%)	19 (28.8%)	0.55^a^
Age 50–65 years (*n* = 242)	74 (30.6%)	80 (33.1%)	0.51^a^
Age 65–80 years (*n* = 361)	154 (42.7%)	163 (45.2%)	0.47^a^
Age > 80 years (*n* = 59)	27 (45.8%)	30 (55.8%)	0.66^a^
*p* value (across age groups)	< 0.001^b^	0.002^b^	

Duration of follow-up was 3.27 ± 1.74 years (range 0.5–7.2 years)

^a^McNemar test

^b^Chi-square test

**Fig. 3:**
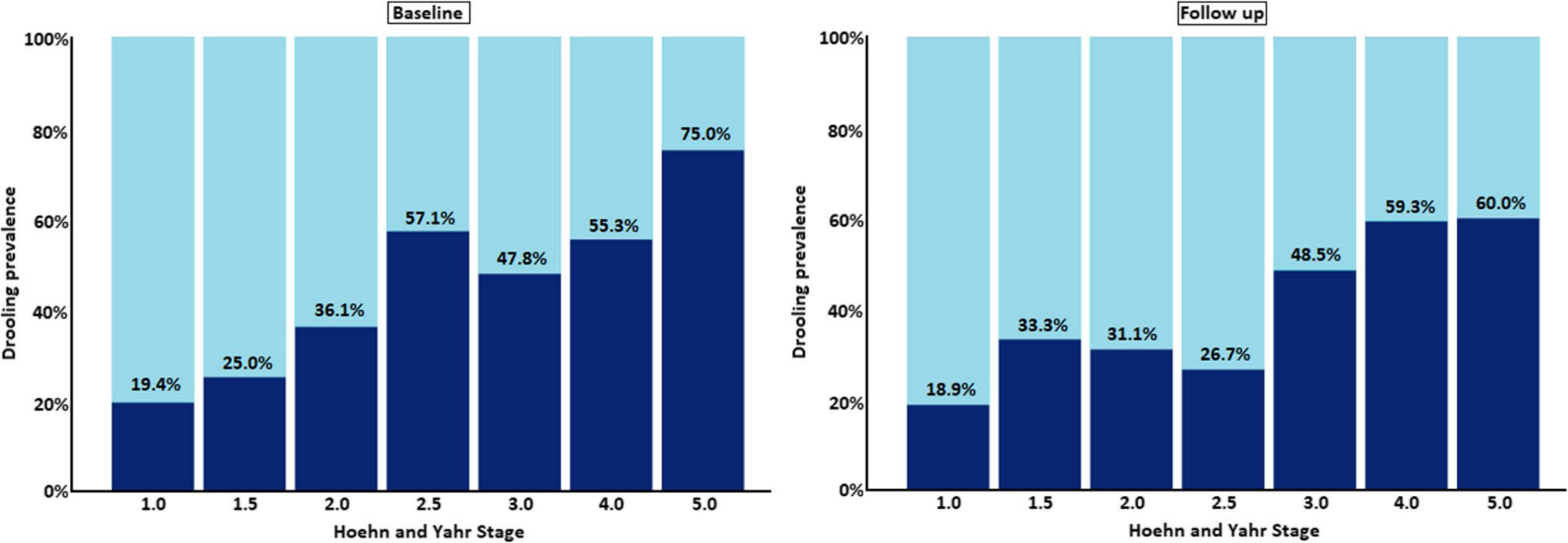
Prevalence of drooling, as defined by question 19 of the Non-Motor Symptoms Scale, at baseline and follow-up across Hoehn and Yahr stages

**Fig. 4:**
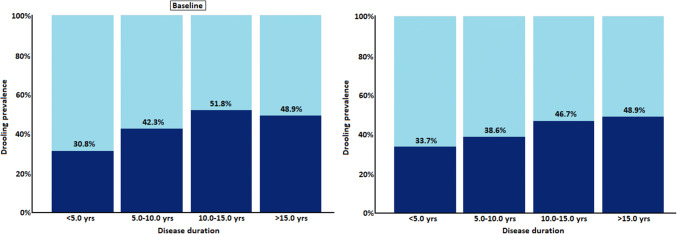
Prevalence of drooling, as defined by question 19 of the Non-Motor Symptoms Scale, at baseline and follow-up across disease duration

When looking at the influence of disease stage, we observed that drooling severity at both baseline and follow-up assessments increased with HY stage (*p* < 0.001 (Wilcoxon signed-rank test), as well as drooling prevalence (McNemar test; *p* < 0.001; Fig. 3). A similar pattern was observed with disease duration, where drooling was most prevalent in in the group of patients with 10.0–15.0 (51.8% and 38.6% at baseline and follow-up, respectively) and > 15.0 years disease duration (48.9% and 48.9% at baseline and follow-up, respectively) (McNemar test; *p* < 0.0.001; Fig. 4).

Of the 456 patients without drooling at baseline, 73 (16.0%) has dysphagia (determined by question 20 of the NMSS), whereas 93 out of 271 (34.3%) PD patients with drooling had dysphagia (Chi-square test; *p* < 0.001). At follow-up, the number of patients with dysphagia had increased, and 20.4% (89 out of 436 patients) with no drooling had dysphagia, and 43.6% (127 out of 291 patients) with drooling had dysphagia.

Both at baseline and at follow-up, drooling severity was significantly, yet almost negligibly, positively associated with PDQ-8 scores (higher scores indicating lower quality of life) (Spearman’s test; ρ =  0.193, *p* < 0.001, and ρ = 0.199, *p* < 0.001, respectively). A regression analysis (*R*^2^ = 0.122; *p* < 0.001) showed that drooling severity (defined by NMSS question 19) at follow-up was predicted by age (*b* = 0.198, *p* < 0.001), MMSE scores (*b* = 0.127, *p* = 0.013), and ESS scores (*b* = 0.142, *p* = 0.012), but not by disease duration, LEDD, HADS subscores, PDSS scores, SCOPA subscores, or NMSS total scores at baseline (*p* ≥ 0.08). The interpretation of this regression model, however, was limited by the very low *R*^2^.

## Discussion

This study represents one of the largest cohorts of PD patients in whom prevalence of drooling was reported, using a validated global NMS scale. Some of the key findings from this large real-life cohort-based data analysis in relation to subjective drooling in PD suggest that drooling occurred in 37.2% in our cohort of PD irrespective of HY stage, disease duration and gender, and that the prevalence of drooling was stable after a follow-up of over 3 years. The latter seems to suggest that treatment was either not initiated or proved ineffective. In addition, drooling appeared to have be associated with patient age, with the highest rates reported in those over 80 years of age. However, drooling severity, as assessed through the NMSS, did not increase over time. Dysphagia may be associated with the presence of drooling, and may need to be specifically considered when determining treatment approaches.

The prevalence of drooling observed in our cohort is similar to that observed in previous studies. Using the Non-Motor Symptom Questionnaire (NMSQ), Erro et al. observed a sialorrhoea frequency of 19.4% in 61 de-novo and untreated patients with PD, increasing to 33.8% after a follow-up of four years [29]. In another study deploying the NMSQ, Picillo and colleagues identified that sialorrhoea in 134 de-novo PD patients increased from 23.3% to 25.0% in men after two years, while it decreased from 10.4 to 4.1% in women [30]. In the most comprehensive study looking at sialorrhoea (defined by the Unified PD Rating Scale drooling item), where 314 PD patients were followed for over 4 years, drooling was present in 11.7% of moderately advanced PD patients, increasing to 55.3% at follow-up [31, 32]. Also, a recent prevalence study on drooling in PD performed in China showed that it is a common problem with the rate of 52.7% (273 out of 518 examined patients). The most common correlations were late onset of disease, higher levodopa equivalent daily doses, higher incidences of dysarthria, dysphagia and fluctuations, higher motor scores, and increased non-motor burden (NMSS) and depression, all affecting quality of life [33]. These studies show, similar to our cohort, that drooling occurs in all stages of PD, and is not an exclusive feature of advanced PD. The differences in prevalence rates may be explained by the use of different (validated) tools to assess siallorhoea and the fact that a large part of patients tend to underreport non-motor symptoms [34].

In our cohort we observed that drooling was often associated with dysphagia, even though it was measured through patient-reported outcomes, and at the last follow-up visits almost half of the patients with drooling had dysphagia. This number is comparable to what was observed in several recent studies. Nienstedt et al. found that in 110 PD patients with a mean disease duration of 9.7 years, half had drooling, and this was associated with dysphagia. In addition, they observed that 59% of patients with severe drooling had dysphagia [35]. Drooling was, as in other studies, also associated with cognitive performance. As drooling appears to occur more often in individuals with PD during cognitively demanding tasks, such as a language task [36], cognitive decline may further contribute to the presence of sialorrhoea. This seems to be supported by observations in other studies showing that drooling in PD is associated with cognitive decline, similar to our findings. Rana and colleagues showed that the presence of dementia, as assessed through the DSM-IV criteria, was significantly associated with the presence of drooling [11]. The link between cognitive performance and drooling in PD, however, could not be confirmed in all studies [33].

To our knowledge, the current study reports the, to date, largest cohort of PD patients in whom sialorrhoea was reported. Despite this, some limitations of our study have to be acknowledged. Firstly, drooling was not assessed through specialist clinical examination, but its presence was assessed in a retrospective cohort. Even though such information may be influenced by e.g. recall bias, the data obtained reflect a real-world experience where similar information is obtained in clinic, where formal assessments are often limited due to time pressure. In addition, a review by the Movement Disorders Task Force on Rating Scales for Parkinson’s Disease suggest the use of NMSQ (recommended) and NMSS (suggested) for global dysautonomia of which drooling and dysphagia form part and NMSS was used in this study [37]. The reason for the NMSS having only a ‘suggested’ recommendation was because of the lack of validation studies other than the original publication, but in the meantime several studies have further validated the NMSS [38–40]. In addition, we did not have specific information on the treatment for drooling, yet the same prevalence of drooling at baseline and follow-up suggests either that drooling was not declared or detected in clinic or ineffectively managed. The latter issue is particularly relevant in light of the recent licencing of Xeomin (Incobotulinumtoxin A) for the management of sialorrhea in PD.

In summary, we observed that subjective sialorrhoea was present in over one-third of participants in a cohort of moderately advanced PD patients, with a stable frequency over a follow-up of over 3 years. These findings confirm those made in previous studies, whereas a novel finding in our study was the effect of age on drooling prevalence in PD. This study underlines the importance of drooling in PD, as well as an urgent need for further studies focusing on appropriate interventions to reduce the prevalence of this bothersome symptom in PD. This is especially relevant as drooling can be socially isolating and embarrassing for PD patients.
